# An Appeal

**Published:** 1922-02

**Authors:** B. Gambier Parry

**Affiliations:** Westhaven, Leckhampton, Cheltenham


*To the Editor of* THE HOSPITAL AND HEALTH REVIEW.

### An Appeal.

*To the Editor of* THE HOSPITAL AND HEALTH REVIEW.

SIR,—May I in your columns state a need of which perhaps your readers are not aware ? Many qualified members of their profession, working on scant salaries and scattered over the most lonely parts of the Empire, have no means of getting up-to-date medical literature. Copies of THE HOSPITAL AND HEALTH REVIEW when finished with would be greatly valued. As secretary for waste medical literature for the S.P.G., I will gladly send an address to anyone undertaking to forward the paper. Old literature and papers sent anonymously cannot be forwarded.—Yours faithfully,

(Miss) B. GAMBIER PARRY.

Westhaven, Leckhampton, Cheltenham.

